# Postprandial Micronutrient Variability and Bioavailability: An Interventional Meal Study in Young vs. Old Participants

**DOI:** 10.3390/nu16050625

**Published:** 2024-02-23

**Authors:** Denny Pellowski, Paula Kusch, Thorsten Henning, Bastian Kochlik, Maria Maares, Amy Schmiedeskamp, Gabriele Pohl, Monika Schreiner, Susanne Baldermann, Hajo Haase, Tanja Schwerdtle, Tilman Grune, Daniela Weber

**Affiliations:** 1 Department Food Chemistry, Institute of Nutritional Science, University of Potsdam, 14469 Potsdam, Germany; 2 NutriAct Competence Cluster Nutrition Research Berlin-Potsdam, 14558 Nuthetal, Germany; 3 Trace-Age-DFG Research Unit on Interactions of Essential Trace Elements in Healthy and Diseased Elderly (FOR 2558), Berlin-Potsdam-Jena-Wuppertal, 14558 Nuthetal, Germany; 4 Department of Molecular Toxicology, German Institute of Human Nutrition Potsdam-Rehbruecke (DIfE), 14558 Nuthetal, Germany; 5 Food4Future (F4F), c/o Leibniz Institute of Vegetable and Ornamental Crops (IGZ), 14979 Grossbeeren, Germany; 6 German Federal Institute for Risk Assessment (BfR), 10589 Berlin, Germany; 7 Department of Food Chemistry and Toxicology, Technische Universität Berlin, 13355 Berlin, Germany; 8 Plant Quality and Food Security, Leibniz Institute of Vegetable and Ornamental Crops, 14979 Grossbeeren, Germany; 9 Faculty of Life Sciences: Food, Nutrition & Health, University of Bayreuth, 95326 Kulmbach, Germany; 10 Department of Physiological Chemistry, Faculty of Chemistry, University of Vienna, 1090 Vienna, Austria

**Keywords:** carotenoids, trace elements, vitamins, postprandial assessment, young, old, interventional meal study

## Abstract

This study explores age- and time-dependent variations in postprandial micronutrient absorption after a micronutrient-rich intervention meal within the Biomiel (bioavailability of micronutrients in elderly) study. Comprising 43 healthy participants, the study compares young (n = 21; mean age 26.90 years) and old (n = 22; mean age 66.77 years) men and women, analyzing baseline concentrations and six-hour postprandial dynamics of iron (Fe), copper (Cu), zinc (Zn), selenium (Se), iodine (I), free zinc (fZn), vitamin C, retinol, lycopene, β-carotene, α-tocopherol, and γ-tocopherol, along with 25(OH) vitamin D (quantified only at baseline). Methodologically, quantifications in serum or plasma were performed at baseline and also at 90, 180, 270, and 360 min postprandially. Results reveal higher baseline serum Zn and plasma lycopene concentrations in the young group, whereas Cu, Se, Cu/Zn ratio, 25(OH) vitamin D, α-tocopherol, and γ-tocopherol were higher in old participants. Postprandial variability of Zn, vitamin C, and lycopene showed a strong time-dependency. Age-related differences in postprandial metabolism were observed for Se, Cu, and I. Nevertheless, most of the variance was explained by individuality. Despite some limitations, this study provides insights into postprandial micronutrient metabolism (in serum/plasma), emphasizing the need for further research for a comprehensive understanding of this complex field. Our discoveries offer valuable insights for designing targeted interventions to address and mitigate micronutrient deficiencies in older adults, fostering optimal health and well-being across the lifespan.

## 1. Introduction

The aging process is linked to changes in nutritional requirements with respect to essential micronutrients. Additionally, alterations in the bioavailability and postprandial variability (linked to the metabolism) of various nutrients may be associated with aging [1]. In this context, the Biomiel (bioavailability of micronutrients in elderly) study emerges as a crucial investigation tool, delving into the postprandial variability and bioavailability of essential trace elements (TEs), vitamins, and carotenoids.

Micronutrients, including the TEs iron (Fe), copper (Cu), zinc (Zn), selenium (Se), and iodine (I), as well as vitamins such as vitamin C, 25(OH) vitamin D, retinol, α-tocopherol, and γ-tocopherol, and the carotenoids lycopene and β-carotene, play crucial roles in diverse biological processes essential for maintaining optimal health, especially as individuals age. Of particular interest with regard to TEs are Zn and Se, recognized for their vital functions in the immune and antioxidant systems and their documented decline in serum levels with aging [2,3]. The bioavailability of Zn typically varies between 20 and 40% and is highly dependent on the source of intake. Studies have shown that the bioavailability of Zn from protein-rich plant-based foods is lower than from protein-rich animal-derived foods. Phytates are also suspected to impair the bioavailability of Zn (and other TEs such as Fe and Cu) [4,5]. Therefore, the German Nutrition Society (DGE) recently decided to link the daily recommended intake of Zn to the daily phytate intake. Postprandially, Zn plays a crucial role in the organism, such as enhancing the insulin signal and gastric acid formation [6], causing strong diurnal fluctuations in serum concentrations [7]. The fraction of free Zn (fZn) seems to be the metabolic active Zn-species [8]. As no study has investigated the postprandial variability of fZn yet, we included it as a potential biomarker for Zn status within this work. Soil Se levels in Germany are frequently deficient; thus, ensuring an adequate dietary provision is not consistently guaranteed. Speciation analysis identified selenomethionine as the major Se compound from food. In healthy individuals, the bioavailability of the organic Se compounds is nearly 100% [9,10]. Se exerts a vital function in the antioxidant system [2]. Similar to Zn, risk groups for developing Se deficiencies are vegans, vegetarians, and the elderly [11,12]. Carotenoids are especially known for their antioxidant properties; they are able to absorb light and are involved in anti-inflammatory processes. Furthermore, some possess pro-vitamin A activity. Thus, they play important roles in the prevention of cancer, cardiovascular diseases, and cognitive disorders [13].

There are indications that lycopene, derived mainly from tomatoes and tomato products, is less bioavailable in older people than other carotenoids such as β-carotene. We previously demonstrated that the plasma concentrations of older participants compared to younger ones are significantly lower in cross-sectional analyses, as seen in the European MARK-AGE Project [14] and the Berlin Ageing Study II (BASE-II) [15]. This inverse association of lycopene and age remained even after adjusting for significant confounders including sex, BMI, season, dietary habits, smoking, and cholesterol, indicating that age is a main factor for plasma lycopene concentrations. Cardinault et al. were able to demonstrate statistically significant differences in the lycopene absorption in eight young vs. eight old men [16]. Therefore, we aim to study the postprandial absorption of different micronutrients, including lycopene, in men and women.

The aging population faces unique challenges related to nutritional status and metabolic dynamics. The alterations in TE-profiles and vitamin levels underscore the need for targeted interventions that address age-related changes in nutrient absorption. The Biomiel study, aimed at contributing to our understanding of healthy aging, addresses this critical gap by comprehensively examining TEs, vitamins, and carotenoids. A distinctive feature of the Biomiel study is its focus on postprandial variability, capturing the dynamic changes in micronutrient serum/plasma concentrations following the consumption of a standardized intervention meal. This approach allows us to shed light on the intricate dynamics of micronutrient metabolism during the postprandial phase in different age groups. The choice of a plant-based test meal, symbolizing health-promoting food innovations, enhances the relevance of our findings for promoting optimal health and well-being in aging individuals.

## 2. Materials and Methods

### 2.1. Subjects and Study Design

The study was approved by the ethics committee of the University of Potsdam, Germany (application no. 72/2020). Informed written consent was obtained from each volunteer. The study was registered under the trial number DRKS00023858 at the German Registry for Clinical Studies.

Participants were recruited through flyers, institutional study newsletter, media announcements, including radio interviews, and in lectures at the University of Potsdam (Germany). Recruitment took place between June 2021 and December 2022.

The primary outcome was the postprandial absorption of different micronutrients in the serum/plasma fraction, whereas secondary endpoints included (for every micronutrient) the maximum serum/plasma concentration, the time at which the maximum concentration was observed, and the difference between both study groups concerning these endpoints, graphically presented as progression curves in the Section 3.

We included healthy women and men between 20–35 and 60–75 years of age, with a BMI between 19 and 29 kg/m^2^ and willingness to abstain from eating foods rich in lycopene, β-carotene, zinc and selenium such as tomatoes, carrots, tomato sauce, ketchup, vegetable juices, sweet potato, pumpkin, persimmon, apricots, papaya, mango, nectarines, peaches, pears, sea buckthorn, kale, spinach, broccoli, arugula, peas, cabbage, corn, Brazil nuts, and wheat germ for three days prior to each intervention day. The detailed exclusion criteria can be found in the Supplemental Materials.

Blood sampling: Fasting blood was collected at baseline and an indwelling cannula placed for further blood draws every 90 min (0, 90, 180, 270, 360 min). Serum (collected in a 2.6 mL vacutainer) and EDTA plasma (collected in a 4.9 mL vacutainer) were prepared by centrifuging blood at 2700× *g* for 10 min. Serum for analyses of TEs and plasma for analyses of carotenoids, tocopherols, retinol, and vitamin C were stored at −80 °C until analyses.

### 2.2. Intervention Meal

The study was conducted as a cross-over study with random allocation to the first intervention meal. The intervention meals consisted of two slices of wheat toast, 10 g margarine, and (a) a micronutrient supplement (lycopene (fair and pure GmbH, Nidda, Germany): 10 mg per capsule; β-carotene (Greenfood Natural Products BV, Sittard, The Netherlands): 30 mg per capsule; participants consumed 1 capsule of the β-carotene supplement and 3 capsules of the lycopene supplement) or (b) a micronutrient-rich spread and a smoothie (see Supplemental Table S1) and the TE content (Supplemental Table S2). The choice of both components is symbolic, representing newly developed foods contributing to health preservation.

There was a washout phase of 14 days between the first and second visit to the study center.

Participants arrived at the study center in an overnight fasting state. After the initial blood sample (baseline), the participants received the intervention meal. Between timepoints of 270 and 360 min, the participants received a sandwich from our institute’s canteen (bread roll, cheese, margarine, few slices of cucumber as decoration), thus being low in carotenoids and TEs.

### 2.3. Determination of Anthropometric Parameters

During the visits, anthropometric measurements such as height, weight, body composition (by bioimpedance analysis), and grip strength were performed at baseline. Furthermore, assessment of skin carotenoids (via reflection spectroscopy, Veggie Meter^®^, Longevity Link Corporation, Salt Lake City, UT, USA) was performed as an average of three scans on the index fingers of both hands, whereas the mean of both hands was used for statistical analysis. The resulting carotenoid reflection score (CRS) was expressed as an arbitrary unit on a scale from 0 to 900. Prior to each measurement, the Veggie Meter^®^ was calibrated using a dark and white reference blank. Furthermore, participants were asked to wash and dry their hands and use hand sanitizer prior to the measurements. After each assessment, the lens of the Veggie Meter^®^ was cleaned with an optical cloth as suggested by the manufacturer. 

### 2.4. Quantification of Trace Elements

The standardized test meal was examined for its TE content. To achieve this, samples of the meal components (approx. 50 mg) first underwent a microwave-assisted acid digestion (Mars6, CEM, Kamp-Lintfort, Germany). The digested solution was diluted 1:10 and afterwards submitted for the quantification of Fe, Zn, Cu, and Se via inductively coupled plasma–tandem mass spectrometry (ICP-MS/MS, Agilent ICP-QQQ-MS 8800, Agilent Technologies, Waldbronn, Germany). The reference materials used were fish muscle (ERM^®^-BB422, European Commission, Joint Research Centre, Geel, Belgium) and pig kidney (ERM^®^-BB186, European Commission, Joint Research Centre, Geel, Belgium). The results of the TE quantification can be found in the Supplemental Table S2.

The quantification of the TEs Fe, Cu, Zn, Se, and I in human serum employed a well-established multi-element approach via ICP-MS/MS [17]. Thawed samples were diluted (1:10) using a solution containing 5 vol.% 1-butanol (Alfa Aesar, Karlsruhe, Germany), 0.05 m% Na-EDTA (Triplex^®^ III, pro Analysis, Merck, Darmstadt, Germany), 0.05 vol.% TritonTM X-100 (10% in H_2_O, Merck-Sigma Aldrich, Steinheim, Germany), and 0.25 vol.% ammonium hydroxide (25% in H_2_O, Fluka, Buchs, Germany) in deionized water. External calibration standards (all elements except Se) were prepared from a 1000 mg/L single-element stock solution (Carl Roth, Karlsruhe, Germany). Rh (5 µg/L, diluted from 1000 mg/L single-element stock solution, Carl Roth, Karlsruhe, Germany) was added as an internal standard. Se quantification utilized isotope dilution analysis. Thus, an internal standard solution containing 50 µg/L Se77 (certified by Trace Sciences International, Ontario, Canada, purchased from Eurisotop SAS, Saarbrücken, Germany) was added. This mixture was directly submitted to analysis via ICP-MS/MS. Additionally, reference sera (ClinCheck^®^ serum Level 1 and 2, RECIPE Chemicals + Instruments, Munich, Germany and SeronormTM, Sero AS, Billingstad, Norway) were included in each analysis. Blank samples (deionized H_2_O) were also subjected to determine the limits of detection (LOD) and limits of quantification (LOQ). We also calculated the Cu/Zn and Se/Cu ratios as they may represent novel biomarkers for aging and various states of disease [18,19,20].

### 2.5. Quantification of Free Zinc

Free Zn as a biomarker for Zn status was quantified in serum samples. Serum concentrations of fZn were assessed utilizing the low-molecular-weight fluorescent sensor Zinpyr-1 (Santa Cruz Biotechnology, Dallas, TX, USA), following the methods described previously [21].

### 2.6. Quantification of Carotenoids and Vitamins

Plasma fat-soluble vitamins A and E as well as the carotenoids were analyzed by reversed-phase HPLC after extraction of lipophilic compounds as previously described [14,22]. Vitamin C was analyzed in plasma supernatants after reduction with tris-(2-carboxyethyl)-phosphine and protein precipitation with metaphosphoric acid solution. Detailed description can be found in [23]. 25(OH) vitamin D was analyzed in plasma via LC-MS/MS as described in detail by Henning et al. [22].

### 2.7. Statistical Analyses

Statistical analyses were conducted using GraphPad Prism Software 10.1.0. Statistical significance was defined as *p* < 0.05, whereas trends were indicated when the *p*-value fell within the range of 0.1 > *p* > 0.05. The normality of distribution was assessed by using the Shapiro–Wilk test. Data exhibiting a normal distribution were examined for significant differences using Student’s *t*-test. For non-normal distributed data Mann–Whitney-U-test was used. To explore the time- and age-dependent trends of the micronutrients in serum/plasma, a repeated measurement two-way ANOVA analysis was utilized. This analysis also allowed us to investigate the explanation of variance in the postprandial variation of the micronutrient concentrations by time, age, and subject. Furthermore, we analyzed the data for significant sex-specific differences at baseline using one-way ANOVA with Tukey’s post hoc test (Supplemental Table S3). However, given the study’s objective to explore age-related variations in postprandial variability, we deliberately formed two age groups (n = 21 vs. n = 22) to facilitate a more comprehensive analysis.

Associations between the baseline micronutrient status and the time-dependent development in the serum/plasma fraction were determined by Pearson correlation coefficient (r). Furthermore, correlations between all measured parameters at baseline were conducted by Pearson correlation coefficient (r). The visualization as a correlation matrix can be found in the Supplemental Figure S1.

## 3. Results

### 3.1. Characteristics of the Study Groups

The mean age (±SD) of the young and old groups was 26.90 years (±3.25) and 66.77 years (±4.73), respectively. Significant differences in anthropometric characteristics were observed between both study groups (Table 1). Young participants demonstrated higher grip strength compared to old participants. Even after normalizing for body mass index (BMI), the young study group exhibited a higher index in grip strength. Parameters associated with body fat were significantly higher in old compared to young participants. In contrast, parameters related to lean body mass were notably higher in the young study group. Moreover, skeletal muscle mass was higher in the young study group. Concerning energy expenditure, significant differences between both groups were only evident in resting energy expenditure. However, total energy expenditure exhibited a tendency to be higher in the young study group. No differences were observed in terms of BMI, total plasma protein levels, and skin carotenoids.

### 3.2. Baseline Status of Micronutrients in Young vs. Old Subjects

**Figure 1:**
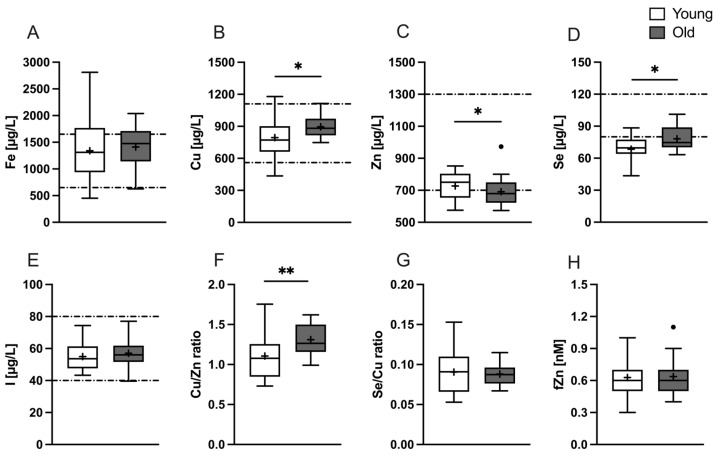
Baseline concentrations of various TEs and TE-associated biomarkers for the young (white) and old (gray) study groups. Data for serum (**A**) Fe, (**B**) Cu, (**C**) Zn, (**D**) Se, (**E**) I, (**F**) Cu/Zn ratio, (**G**) Se/Cu ratio, and (**H**) fZn are plotted as a box-and-whiskers plot with straight lines: median; +: mean; •: identified outlier. The dashed lines represent the reference ranges (if available). Significant differences are marked with *p* < 0.05 (*); *p* < 0.01 (**) and were calculated by unpaired *t*-test.

At baseline, old participants had significantly higher serum Cu and Se concentrations (895.4 ± 102.3 µg/L vs. 794.5 ± 182.7 µg/L, *p* = 0.030 and 78.21 ± 11.01 µg/L vs. 68.44 ± 12.65 µg/L, *p* = 0.025, respectively, as depicted in Figure 1B,D) compared to young participants. The mean serum Se concentration was below the reference range in both groups. Serum Zn concentrations in the young study group fell within the lower reference range, while insufficient concentrations were observed for the majority of old participants. The difference between both groups was identified as significant (young: 727.2 ± 86.35 µg/L vs. old: 678.1 ± 65.18 µg/L, *p* = 0.044; Figure 1C). The calculated Cu/Zn ratio was significantly higher in the old compared to the young study group (1.311 ± 0.199 vs. 1.106 ± 0.291, *p* = 0.0099; Figure 1F). No differences were observed between both groups for Fe (young: 1342 ± 561.4 µg/L vs. old: 1412 ± 368.1 µg/L, *p* = 0.633; Figure 1A), I (young: 54.94 ± 8.41 µg/L vs. old: 57.02 ± 8.18 µg/L, *p* = 0.422; Figure 1E), Se/Cu ratio (young: 0.091 ± 0.027 vs. old: 0.088 ± 0.014, *p* = 0.712; Figure 1G), and fZn (young: 0.629 ± 0.149 nM vs. old: 0.636 ± 0.173, *p* = 0.875; Figure 1H).

**Figure 2:**
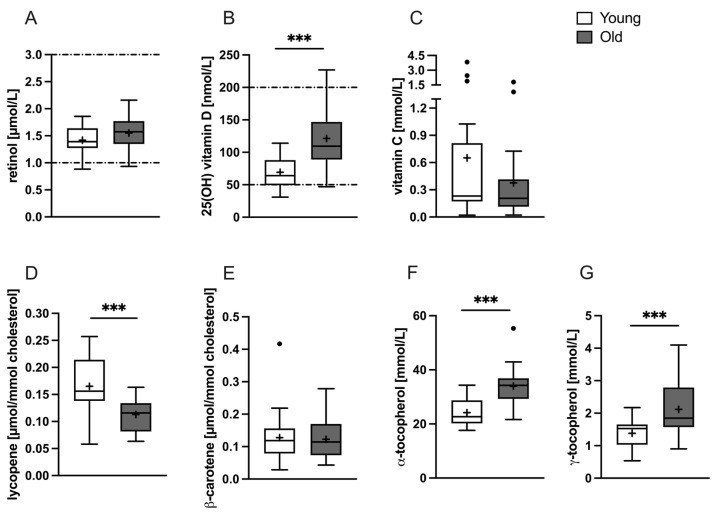
Baseline concentrations of various vitamins and carotenoids for the young (white) and old (gray) study groups. Data for plasma (**A**) retinol, (**B**) 25(OH) vitamin D, (**C**) vitamin C, (**D**) normalized lycopene, (**E**) normalized β-carotene, (**F**) α-tocopherol, and (**G**) γ-tocopherol is plotted as box-and-whiskers plot with straight lines: median; +: mean; •: identified outlier. The dashed lines represent the reference ranges (if available). Significant differences are marked with *p* < 0.001 (***) and were calculated by unpaired *t*-test.

25(OH) vitamin D, α-tocopherol, and γ-tocopherol levels were significantly higher in old, compared to young participants (121.3 ± 48.68 nmol/L vs. 69.19 ± 26.87 nmol/L, *p* = 0.0001, Figure 2B; 32.96 ± 5.18 mmol/L vs. 24.16 ± 5.06 mmol/L, *p* < 0.0001, Figure 2F; and 2.12 ± 0.84 mmol/L vs. 1.38 ± 0.42 mmol/L, *p* = 0.0004, Figure 2G, respectively). Conversely, the mean of lycopene normalized to cholesterol was significantly higher in young participants (0.165 ± 0.048 µmol/mmol cholesterol vs. 0.113 ± 0.029 µmol/mmol cholesterol, *p* < 0.0001; Figure 2E). No differences were observed for retinol (1.419 ± 0.256 µmol vs. 1.552 ± 0.301 µmol, *p* = 0.134; Figure 2A), cholesterol-normalized β-carotene (0.113 ± 0.051 µmol/mmol cholesterol vs. 0.123 ± 0.060 µmol/mmol cholesterol, *p* = 0.575; Figure 2F), and vitamin C (0.306 ± 0.293 mmol/L vs. 0.255 ± 0.199 mmol/L, *p* = 0.784; Figure 2C).

### 3.3. Postprandial Variability of Micronutrients

**Figure 3:**
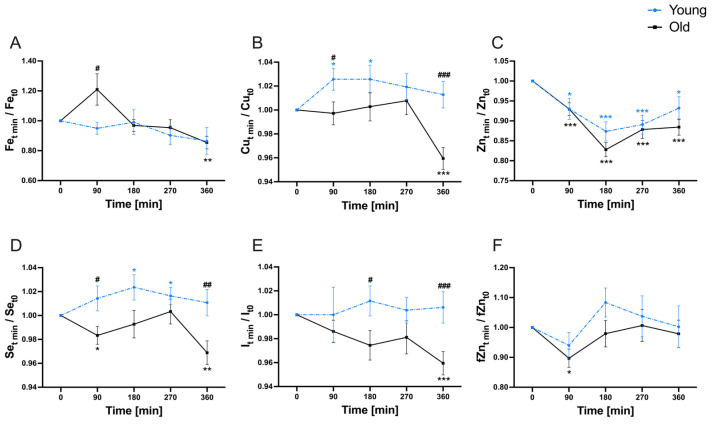
Postprandial progression curves of serum (**A**) Fe, (**B**) Cu, (**C**) Zn, (**D**) Se, (**E**) I, and (**F**) fZn concentrations throughout 360 min after consumption of the micronutrient-rich meal within the young (dashed blue) and old (black) study groups. Shown are the mean (±SEM) ratios of the serum concentrations between each observed timepoint and baseline to reflect postprandial variability. Significant time-dependent differences are marked with *p* < 0.05 (*); *p* < 0.01 (**); *p* < 0.001 (***). Significant differences that were age-related are marked with *p* < 0.05 (#); *p* < 0.01 (##); *p* < 0.001 (###). Calculations for significance were performed using repeated measurement two-way ANOVA followed by Fisher’s LSD post hoc test.

**Figure 4:**
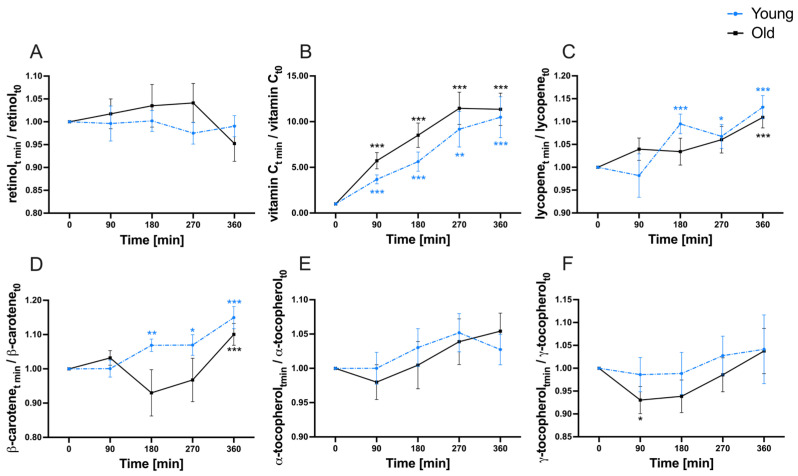
Postprandial progression curves of plasma (**A**) retinol, (**B**) vitamin C, (**C**) normalized lycopene, (**D**) normalized β-carotene, (**E**) α-tocopherol, and (**F**) γ-tocopherol throughout 360 min after consumption of the micronutrient-rich meal within the young (dashed blue) and old (black) study groups. Shown are the mean (±SEM) ratios of the plasma concentrations between each observed timepoint and baseline to reflect postprandial variability. Significant time-dependent differences are marked with *p* < 0.05 (*); *p* < 0.01 (**); *p* < 0.001 (***). Calculations for significance were performed using repeated measurement two-way ANOVA followed by Fisher’s LSD post hoc test.

In addition to baseline assessment, quantifications of the investigated micronutrients were performed at 90, 180, 270, and 360 min (postprandial) to explore the variability of micronutrients in the serum and plasma fraction, respectively (Figure 3 and Figure 4).

Serum Fe displayed a significant decline in the old study group by the end of the observation period compared to baseline concentrations, while no time-dependent disparity was noted in the young study group (Figure 3A). An age-dependent variation between both groups was evident at 90 min. The progression curves of serum Cu (Figure 3B), serum Se (Figure 3D), and serum I (Figure 3E) exhibited generally similar patterns. In the young study group, Cu was significantly elevated after 90 min following meal consumption. However, by the end of the observation period, no significant alteration in serum Cu concentrations was observed in comparison to the baseline. Conversely, the old participants did not demonstrate changes in Cu serum concentrations until 270 min after the test meal. After 360 min, a significant decrease in Cu was noted compared to the baseline. Age-dependent distinctions were observed at timepoints of 90 min and 360 min. Selenium increased significantly to a serum peak at timepoint 180 min within the young study group. By the end of the investigation, no variance was identified in relation to baseline concentrations. In the old study group, serum Se were significantly decreased after 90 min and again after 360 min. Age-related differences were observed at 90 min and also at 360 min. Iodine concentrations displayed no significant time-dependent fluctuations within the young study group. However, a significant decrease in serum I within the old participants was noted after 360 min. Age-based differences were observed at the timepoints 180 min and 360 min. Serum Zn concentrations demonstrated a strong time-dependency (Figure 3C). Within the initial 180 min after meal consumption, serum Zn concentrations significantly decreased to a minimum. Subsequently, serum Zn concentrations gradually increased without reaching baseline levels. No age-dependencies were observed in the postprandial variability of Zn concentrations in the serum fraction. For fZn, there was a decline within the first 90 min after food consumption, which was significant only within the old study group (Figure 3F). Subsequently, fZn exhibited an increase without notable differences compared to the baseline at the end of the investigation period. No age-related differences were found in the postprandial variability of the fZn fraction in serum.

A multi-fold increase compared to baseline was observed for vitamin C in both study groups, whereas no age-related differences were found (Figure 4B). The carotenoids lycopene (Figure 4C) and β-carotene (both normalized to cholesterol) (Figure 4D) exhibited a comparable pattern in the variability within the plasma after test meal consumption. In both study groups, both micronutrients reached the highest observed concentration by the end of the investigation period (360 min). This increase differed significantly from baseline levels in both groups. Postprandial variability of γ-tocopherol showed no age-specific differences (Figure 4F). A significant decrease in plasma γ-tocopherol compared to baseline was observed within the old study group after 90 min. In contrast, plasma retinol and α-tocopherol displayed no discernable time- or age-dependent differences throughout the observation period (Figure 4A,E).

The postprandial progression curves of plasma after the intake of a lycopene and β-carotene supplement are shown in a Supplemental Figure S2.

All parameters displayed the highest percentage of the total variance explained by the individual factor (Table 2). The variable “subject” accounted for 30.84% (Fe), 31.41% (Cu), 40.33% (Zn), 28.45% (Se), 31.77% (I), 56.14% (fZn), 61.64% (retinol), 39.00% (lycopene), 35.51% (β-carotene), 45.16% (α-tocopherol), and 49.95% (γ-tocopherol of the total variance. Significant percentages of total variance were observed concerning time for Fe (8.01%), Cu (6.51%), Zn (28.49%), fZn (4.28%), lycopene (15.16%), β-carotene (8.33%), and α-tocopherol (4.46%). The factor age exhibited notable percentages of the total variation for Cu (7.60%), Se (9.24%), and I (6.20%).

### 3.4. Relationship between Baseline Status and Postprandial Variability of Micronutrients in Serum/Plasma

**Figure 5:**
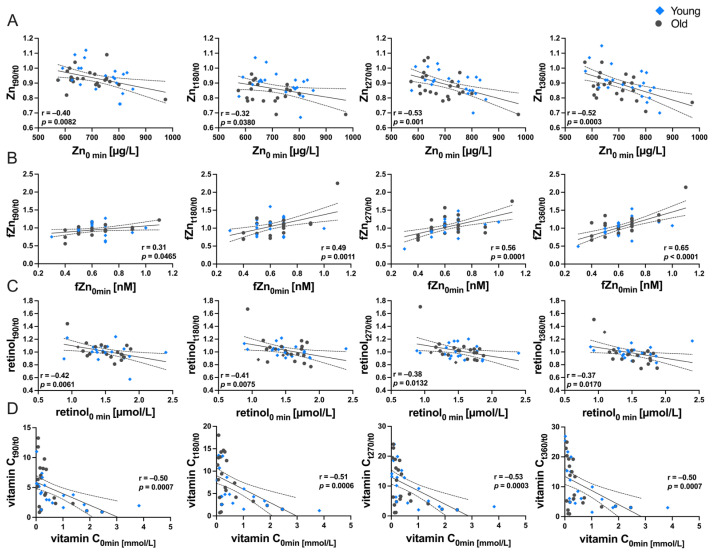
Correlations between baseline serum/plasma concentrations and ratios of sampling timepoints to investigate the impact of the baseline status on the postprandial variability of (**A**) Zn, (**B**) fZn, (**C**) retinol, and (**D**) vitamin C in serum/plasma throughout a 360 min investigation period. The blue diamonds indicate young participants, while the gray circles represent old participants. Correlation was determined by Pearson correlation coefficient (r), provided with the corresponding *p*-values. Additionally, 95% confidence intervals are provided.

Correlation analyses were conducted to examine the association between the baseline concentration and the postprandial variability for each micronutrient (Figure 5). Our findings revealed a significant negative correlation between baseline serum Zn concentrations and its postprandial changes. This association was significant at every investigated timepoint (Figure 5A). In contrast, the fZn pool in serum displayed a positive correlation between baseline concentration and changes postprandial changes (Figure 5B).

Moreover, negative associations between baseline plasma concentration and changes after food consumption were observed for retinol (Figure 5C). Eventually, the investigations on vitamin C revealed significant inverse correlations between baseline concentrations and postprandial variability. 

Other micronutrients exhibited significant correlations at individual timepoints only (Fe: 270 min; Se: 270 min; β-carotene: 360 min; γ-tocopherol: 360 min). No correlations between baseline concentrations and postprandial changes were observed for Cu, I, lycopene, and α-tocopherol (Table 3). Results for the supplement intake are not shown.

## 4. Discussion

The objective of this study was to explore age- and time-dependent differences in the postprandial variability of various micronutrients. This involved examining baseline concentrations and the variability of micronutrients throughout a 360 min observation period. Additionally, we assessed other factors influencing the postprandial variability of micronutrients in serum/plasma, such as individuality and baseline status.

Our results uncovered higher baseline serum Zn concentrations in the young compared to old participants, which is consistent with the previous literature [3]. The mean serum Zn concentrations within the young study group were slightly above the lower reference limit [24], while those in the old group fell below the reference range, indicating widespread inadequacy within this age-group. Postprandial serum variability showed a significant decrease in the first 180 min after test meal consumption, followed by a gradual re-increase without reaching pre-prandial levels. Given Zn’s role in digestive processes such as enhancing the insulin signal [25], the observed postprandial decline may reflect systemic distribution from serum to tissues. The process appears dependent on time and the individual factor. Baseline status also proved relevant to postprandial variability, with participants with lower baseline concentrations tending to reach postprandial concentrations above that level within the observation period. The literature stated that the bioavailability of Zn from different food sources may be reduced in an adequate supply state [6]. This may be justified by the larger available Zn pool in the serum fraction which, consequently, can be “drawn” for metabolic and digestive processes. For testing these hypotheses further analyses are necessary.

In contrast to total Zn in serum, no age-specific differences were observed concerning the fZn fraction in serum at baseline. To our best knowledge, there is only one study to investigate age-related differences in fZn serum concentrations in humans [21]. However, no age-related changes in fZn were also observed. Another recent study exhibited no age-specific differences in mice [26]. Additionally, the postprandial variability of fZn was not studied before. Similar to total Zn, we observed a decline in the fZn fraction in serum shortly after food intake, which was only significant within the old study group. The fZn concentration showed a tendency to re-increase afterwards by keeping a stable level by then, suggesting a compensatory mechanism. Further research will be needed in order to acquire insights into the biological functions of the fZn species in serum.

The old participants exhibited higher baseline serum Se concentrations, potentially due to a higher number of vegans and vegetarians among the young study group. However, both study groups had mean Se baseline serum concentrations below the reference range, indicating inadequacy. Postprandial changes in Se concentrations revealed age- and time-dependent differences. Various articles focus on the long-term bioavailability of Se [27,28]. However, studies on the short-term and especially postprandial bioavailability of Se are scarce. It is important to mention that Se metabolism is highly complex. Repeated measurements of Se in serum might also reflect the postprandial variability of selenoprotein P and other selenoproteins. Another study examined Se absorption from food by calculating differences between intake and retention (feces and urine) [29]. Our results involve screening all selenium species in serum and only provide limited insights into bioavailability. Nevertheless, there appear to be age-related differences in postprandial Se metabolism.

Higher Cu concentrations at baseline among the old participants align with findings in other studies. Highly variable Cu concentrations in serum are mostly indicative of an impaired health status [30]. Thus, serum Cu is well-regulated in healthy individuals. After uptake by the enterocytes and secretion into the bloodstream, Cu is transported to the liver by various chaperones, including albumin and transcuprein. Our results may reflect the Cu metabolism in the serum. Regarding the young study group, a significant increase in serum Cu concentrations was observed 90 min after food intake, which may reflect the absorption/secretion phase. The Cu concentrations gradually decreased afterwards, which may indicate the hepatic uptake. With regards to the old study group, there was a more drastic decline of serum Cu concentrations when comparing the timepoints 270 min and 360 min. This may also be due to elimination processes of Cu from the serum. Further research could provide a better understanding of postprandial Cu variability.

In terms of the potential novel biomarkers Cu/Zn ratio and Se/Cu ratio, we only observed significant age-specific differences in the former, indicating that this marker might serve as a more sensitive biomarker specifically for aging in our study design. The regional EPIC-Potsdam cohort, however, found differences with aging in both parameters [3].

No age-related differences were found in baseline serum Fe concentrations. However, it is still under debate as to whether serum Fe is a representative biomarker for evaluating the Fe status [31]. Other studies that investigated changes of serum Fe with aging observed increased concentrations within the elderly population [3]. Another study considering serum Fe concentrations additionally found that an overnight fasted state was associated with higher Fe in serum. The same study observed a noticeable stability of serum Fe throughout a 21 h collection period [32]. An alternative study demonstrated increased serum Fe after test meal consumption only for participants dealing with haemochromatosis or iron deficiency anemia, while the Fe serum concentrations of healthy individuals were nearly unaffected [33]. Our results also exhibited an overall stability throughout a 6 h collection window within the young study group. However, even for postprandial serum Fe concentrations, the intra-individuality seems to play a role, as our results reveal. Old participants seem to be more sensitive to diurnal variations in Fe concentrations, as we examined a significant difference between baseline and 360 min postprandial serum concentrations.

Baseline serum I concentrations showed no significant differences between either study group. Also, regarding I, the regional EPIC-Potsdam cohort revealed an increase in serum concentrations with aging [3]. No significant postprandial changes were observed within the young study group. In contrast, a significant decline in serum I concentrations was found within the old study group by the end of the observation period. This could be due to variations in postprandial thyroid hormone metabolism, as those are mainly quantified when measuring I in serum. These variations could affect the diurnal rhythm of thyroid hormones in aging. Reduced hormonal activity, including alterations in the hypothalamic–pituitary–thyroid axis and coupled with a decline in metabolic activity, is also conceivable [34]. Anthropometric analysis revealed a significant difference in basal metabolic rate between the two study groups. Medications influencing thyroid hormone metabolism, such as amiodarone or glucocorticoids, are also a possible explanation for our observations.

In our analysis of sex-specific differences, we observed that significant variations were evident only in baseline serum Zn concentrations, and specifically between young males and females. Notably, all other TEs or TE-associated markers, including fZn, Cu/Zn ratio, and Se/Cu ratio, exhibited no discernible sex-specific differences (as indicated in Supplemental Table S3). This underscores the robustness of our study design in ensuring comparability, particularly in terms of group size, and emphasizes the primary focus on investigating age-related differences in postprandial variability.

It is crucial to highlight that, concerning the relative postprandial variability of trace elements, no sex-specific differences were observed.

Baseline retinol was within the normal range (1.0–3.0 µmol/L) in both study groups (1.4–1.5 µmol/L) [35]. Plasma retinol is under homeostatic regulation in healthy individuals and no retinol was added to the intervention meal. Thus, we did not observe any differences after test meal consumption in young or old participants, and no sex-related differences. Baseline retinol status, however, did impact postprandial retinol levels significantly.

Margarine, derived from sunflower oil, comprises approximately 6 mg of α-tocopherol and 10 mg of γ-tocopherol per 100 g [36]. Consequently, the administered dosage to the participants was approximately 1.2 mg and 2 mg, respectively. As anticipated, there was no discernible increase in either α- or γ-tocopherol levels from these administered doses throughout the 6 h observation period.

25(OH) vitamin D levels were significantly higher in the old study group, likely due to supplement intake (not shown). 25(OH) vitamin D does not exhibit a notable response to this brief period owing to its extended biological half-life of 2–3 weeks [37]. Since 25(OH) vitamin D was not a part of the intervention meal, we did not further focus on it within the scope of the study.

Baseline vitamin C was within the normal range of around 0.3 mmol/L [38] without significant age- or sex-related differences. The consumption of the test meal led to a significant increase in both study groups, without revealing significant age-related differences. The factors affecting vitamin C plasma concentration seem to be time and the subject, showing that age is not associated with plasma vitamin C. Baseline vitamin C status showed an inverse correlation with ratios of sampling timepoints.

Baseline plasma lycopene was lower in the old group, as expected according to our previous findings [14,15]. It increased significantly during the observation period. Age was not significantly associated with plasma lycopene variance, but time and interindividuality significantly affected postprandial lycopene variability (15% and 39% of variation, respectively).

Concerning differences related to sex, we only observed higher concentrations in the old men compared to the old women.

β-Carotene showed an age-related difference only in females at baseline (higher levels in the young women). The literature suggests higher plasma β-carotene in women compared to men. In the present study, this was only the case in the young group (Supplemental Table S3). β-Carotene increased significantly during the six-hour observation period but with no difference between the study groups. Age was not significantly associated with plasma β-carotene variance, but time and subject significantly affected postprandial β-carotene variability. The only observed correlations between baseline concentrations and postprandial changes were observed for 360 min.

There are some limitations to consider. The duration of blood sampling was not long enough for thorough analyses of postprandial carotenoid bioavailability; a duration of 8–9 h would have been favorable. We were not able to carry out separation of the chylomicron/lipoprotein fraction, which could have resulted in earlier peaks, especially for carotenoids. Due to the COVID-19 pandemic, the study duration was much longer than anticipated (18 months), which additionally resulted in difficulty recruiting participants due to the “medical environment” and exposure to other people. The older participants in particular were rather health-oriented, which might have resulted in selection bias.

The ingestion of capsules containing each 30 mg lycopene and β-carotene resulted only in minimal changes in plasma lycopene and β-carotene. This is likely due to the fact that the supplement matrix consisted of microcellulose (HPMC, hydroxypropyl methylcellulose), which is soluble in water. However, the supplements were provided with toast and margarine for better comparison with the micronutrient-rich intervention meals. It has been shown that after 120 min at gastric pH, the water-soluble polymer is dissolved only to 69 ± 17% [39]. Therefore, for the supplements with HPMC matrix a longer sampling time would be beneficial. To the best of our knowledge, this is the first study assessing the short-term postprandial progression of carotenoids from microcellulose capsules; therefore, we cannot compare our results to previous work.

Eventually, it is essential to acknowledge the notable strengths that contribute to the robustness and significance of our findings. The Biomiel study provides a comprehensive approach to investigating age- and time-dependent differences in postprandial micronutrient absorption, a relatively understudied aspect of nutritional science in aging populations. The meticulous assessments, including anthropometric measurements, grip strength evaluations, and skin carotenoid assessments, offer a detailed overview of participants’ nutritional and physiological status. The focus on a plant-based test meal, comprising a micronutrient-rich spread and smoothie, adds a novel dimension by symbolizing health-promoting food innovations. Furthermore, the inclusion of both young and old age groups enhances the generalizability of our findings and provides valuable insights into the dynamics of postprandial micronutrient metabolism across different life stages.

Our results indicate that the postprandial variability of the micronutrients observed is highly complex. The most significant influencing factor is the interindividual variability. Nevertheless, a residual variability of 30–60% remains for the micronutrients investigated here. We were able to elucidate that the baseline status of Zn is associated with postprandial variability. The same holds true for fZn, retinol, and vitamin C. Since the baseline status also contributes to the individual factor, the question remains open as to which proportion of the residual variation can be explained by the baseline status. Further research regarding postprandial variability is necessary in order to gain a deeper understanding of this complex research field. A higher number of study participants, as well as additional examination of urine and feces samples would undoubtedly provide further insights into bioavailability. Extending the sampling period would also be conceivable and could help to gain insight into the all-day variability of micronutrients comparing young and old individuals. Our results provide a basic idea of how the metabolism of various micronutrients changes after the intake of a plant-based and standardized test meal in young vs. old participants. We identified various factors likely to influence postprandial variability, including individuality, age, and time. Our findings could have important implications for the development of targeted interventions to prevent and manage micronutrient deficiencies in older adults and to promote optimal health and well-being throughout the lifespan.

## 5. Conclusions

In this study, we have explored the use of innovative food interventions for investigations into nutrient absorption, providing valuable insights into postprandial micronutrient variability. The utilization of a plant-based test meal, comprising a micronutrient-rich spread and smoothie, symbolizes a novel approach in nutritional science for aging populations.

A notable contribution of this research is the examination of fZn as a potential biomarker for postprandial serum Zn status. To our knowledge, this is the first instance of such an investigation, shedding light on the dynamics of Zn metabolism after food intake. The findings suggest that fZn may serve as a sensitive indicator of Zn status, offering a new dimension to the assessment of Zn adequacy in nutritional studies.

Our study stands out by concurrently screening multiple nutrients within a bioavailability framework, allowing for a comprehensive evaluation of the postprandial variability of various micronutrients from a test meal. This approach provides a more holistic understanding of how different nutrients respond to a standardized intervention, offering a nuanced perspective on their absorption and metabolism.

We included both men and women in our analyses; however, sex does not seem to have a major impact on the results in our selected study population. Furthermore, the comparison between older and younger age groups provides a comprehensive overview across different life stages. This age-stratified analysis enhances the generalizability of our findings and contributes to the existing knowledge gap in postprandial nutrient absorption, especially in aging populations. The observed age-dependent differences in baseline Cu, Zn, Se, vitamin D, lycopene, α-tocopherol, and γ-tocopherol concentrations underscore the importance of considering age-related factors in designing interventions and formulating nutritional recommendations for diverse age groups.

## Figures and Tables

**Table 1:** Descriptive characteristics of the study participants at baseline.

Parameter	Young (n = 21)	Old (n = 22)	*p*
Age [Years]	26.90 ± 3.25	66.77 ± 4.73	<0.0001 ^a^
sex, female [n (%)]	12 (57)	15 (68)	0.4540 ^b^
Vegan/vegetarian [n (%)]	7 (33)	4 (18)	0.2550 ^b^
BMI [kg/m^2^]	24.02 ± 2.73	24.73 ± 3.21	0.4350 ^c^
Grip strength [kg]	41.79 ± 13.54	33.91 ± 10.80	0.0220 ^a^
Grip normalized ^1^	1.73 ± 0.46	1.37 ± 0.37	0.0074 ^a^
Absolute fat mass [kg]	18.94 ± 4.62	23.33 ± 6.85	0.0184 ^c^
Relative fat mass [%]	25.57 ± 6.00	32.75 ± 6.90	0.0008 ^c^
FMI	5.90 ± 1.26	8.18 ± 2.23	0.0002 ^c^
Lean body mass [kg]	55.64 ± 10.69	48.00 ± 11.55	0.0074 ^a^
LBMI	17.85 ± 2.34	16.55 ± 2.18	0.0448 ^a^
Skeletal muscle mass [kg]	26.50 ± 6.35	21.68 ± 6.69	0.0036 ^a^
Total energy expenditure [kcal]	2650 ± 412	2440 ± 472	0.0756 ^a^
Resting energy expenditure [kcal]	1628 ± 228	1433 ± 245	0.0028 ^a^
Total plasma protein [mg/mL]	74.98 ± 7.44	71.90 ± 6.45	0.1531 ^c^
Skin Carotenoids	386.6 ± 83.2	387.0 ± 92.4	0.9882 ^c^

BMI: body mass index; FMI: fat mass index; LBMI: lean body mass index. ^1^ to BMI. ^a^ Mann-Whitney-U-test. ^b^ Chi-Squared test. ^c^ Unpaired *t*-test.

**Table 2:** Explanation of variance in the postprandial variability of micronutrient concentrations by time, age, and subject.

Micronutrient	% of Total Variation			*p*		
	Time	Age	Subject	Time	Age	Subject
Fe	**8.01**	1.16	**30.84**	**0.0055**	0.2967	**0.0010**
Cu	**6.51**	**7.60**	**31.41**	**0.0137**	**0.0115**	**0.0002**
Zn	**28.49**	1.29	**40.33**	**<0.0001**	0.3360	**<0.0001**
Se	3.54	**9.24**	**28.45**	0.1395	**0.0040**	**0.0028**
I	1.29	**6.20**	**31.77**	0.5676	**0.0242**	**0.0015**
fZn	**4.28**	0.02	**56.14**	**0.0341**	0.9140	**<0.0001**
retinol	1.49	0.40	**61.64**	0.2800	0.6619	**<0.0001**
vitamin C	**30.82**	1.50	**43.81**	**<0.0001**	0.3198	**<0.0001**
lycopene (normalized)	**15.16**	0.08	**39.00**	**<0.0001**	0.8014	**<0.0001**
β-carotene (normalized)	**8.33**	0.11	**35.51**	**0.0046**	0.1577	**<0.0001**
α-tocopherol	**4.46**	0.10	**45.16**	**0.0424**	0.8005	**<0.0001**
γ-tocopherol	3.61	0.92	**49.95**	0.0824	0.4705	**<0.0001**

The table presents the results for the source of variation of the conducted repeated measurements two-way ANOVA. Bold type indicates significance (*p* < 0.05).

**Table 3:** Pearson correlations between baseline micronutrient concentrations and ratios of sampling timepoints and baseline.

Micronutrient at Baseline	t_90_/t_0_		t_180_/t_0_		t_270_/t_0_		t_360_/t_0_	
	r	*p*	r	*p*	r	*p*	r	*p*
Fe	−0.29	0.0977	−0.32	0.0721	**−0.46**	**0.0074**	−0.34	0.0532
Cu	−0.26	0.1514	−0.23	0.1906	−0.08	0.6525	−0.14	0.4372
Zn	**−0.40**	**0.0082**	**−0.32**	**0.0380**	**−0.51**	**0.0005**	**−0.52**	**0.0003**
Se	−0.15	0.4156	−0.27	0.1317	**−0.50**	**0.0028**	−0.03	0.8803
I	0.03	0.8926	0.02	0.8985	−0.03	0.8626	−0.21	0.2455
fZn	**0.31**	**0.0465**	**0.49**	**0.0011**	**0.56**	**0.0001**	**0.65**	**<0.0001**
retinol	**−0.42**	**0.0061**	**−0.41**	**0.0075**	**−0.38**	**0.0132**	**−0.37**	**0.0170**
vitamin C	**−0.50**	**0.0007**	**−0.51**	**0.0006**	**−0.53**	**0.0003**	**−0.50**	**0.0007**
lycopene normalized	−0.26	0.1403	0.29	0.1084	0.06	0.7274	−0.02	0.9257
β-carotene normalized	−0.02	0.9064	−0.14	0.4582	−0.24	0.1710	**−0.47**	**0.0063**
α-tocopherol	−0.30	0.0900	0.15	0.4095	−0.21	0.2340	−0.18	0.3215
γ-tocopherol	−0.12	0.5297	−0.07	0.6958	−0.19	0.2960	**−0.52**	**0.0021**

Bold type indicates significance (*p* < 0.05).

## Data Availability

Data are contained within the article and Supplementary Materials.

## Supplementary Materials

The following supporting information can be downloaded at https://www.mdpi.com/article/10.3390/nu16050625/s1, Figure S1: Correlation matrix for parameters, investigated within the Biomiel study; Figure S2: Postprandial progression curves of (A) lycopene and (B) β-carotene plasma concentrations throughout 360 min after consumption of a carotenoid-enriched supplement within the young (blue) and old (gray) study groups; Table S1: Ingredients and quantities for the test meal; Table S2: Trace element concentrations for the test meal components and corresponding percentage proportions of the DRI according to the German Nutrition Society; Table S3: Sex-specific differences in examined parameters provided as mean ± SD; Material S1: Exclusion criteria.
