# Supplementary material for: Postprandial Micronutrient Variability and Bioavailability: An Interventional Meal Study in Young vs. Old Participants
Source: Nutrients. 2024 Feb 23;16(5):625. doi: 10.3390/nu16050625 (PMC10934810; doi:10.3390/nu16050625)
Supplement: Supplementary file 1 [file nutrients-16-00625-s001.zip › nutrients-2853082-supplementary.pdf]

Supplemental Figure S1

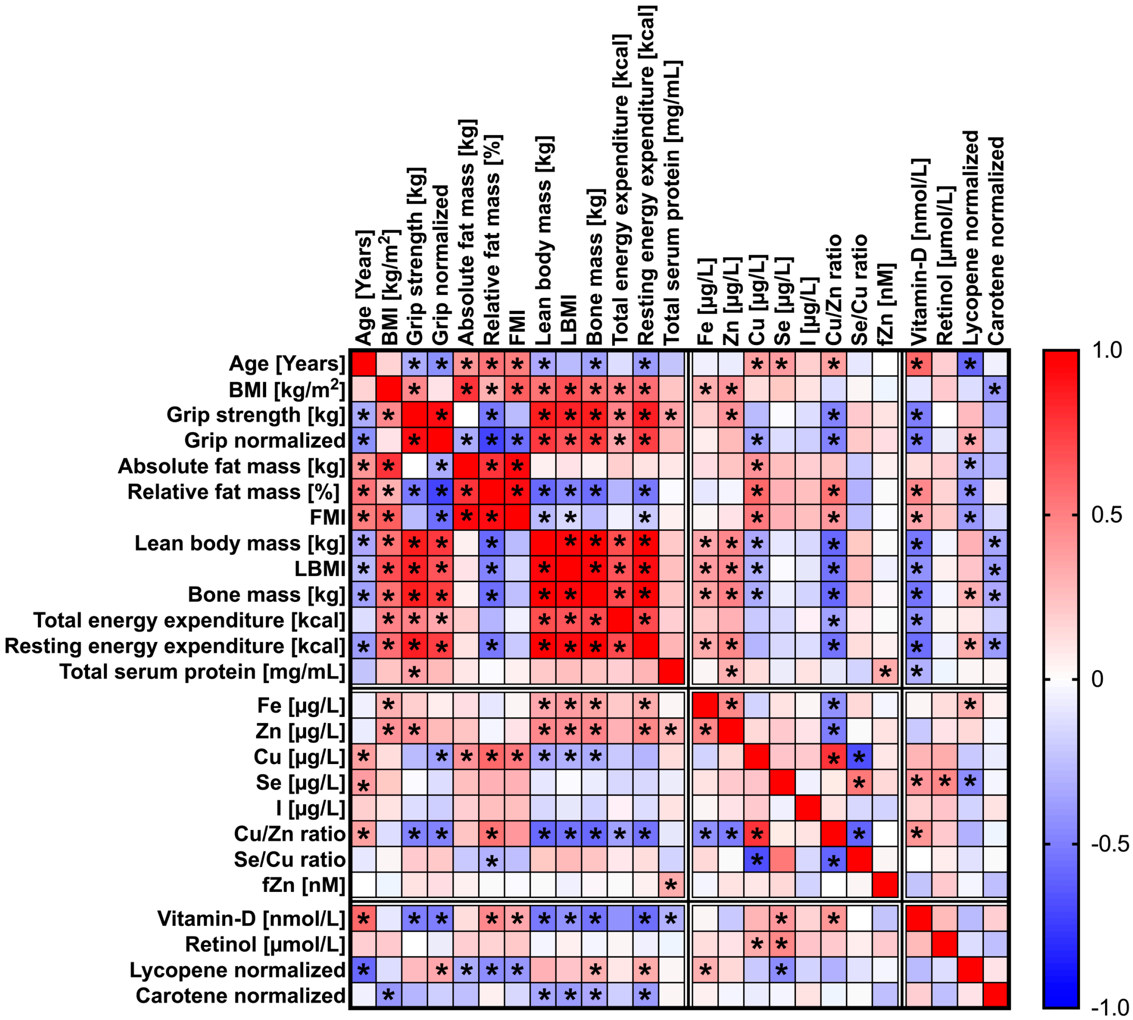

Figure S1. Correlation matrix for parameters, investigated within the Biomiel study. Positive associations are indicated in red. The blue color indicates negative associations. Significant correlations are marked by an asterisk ($p < 0.05$).

Supplemental Figure S2

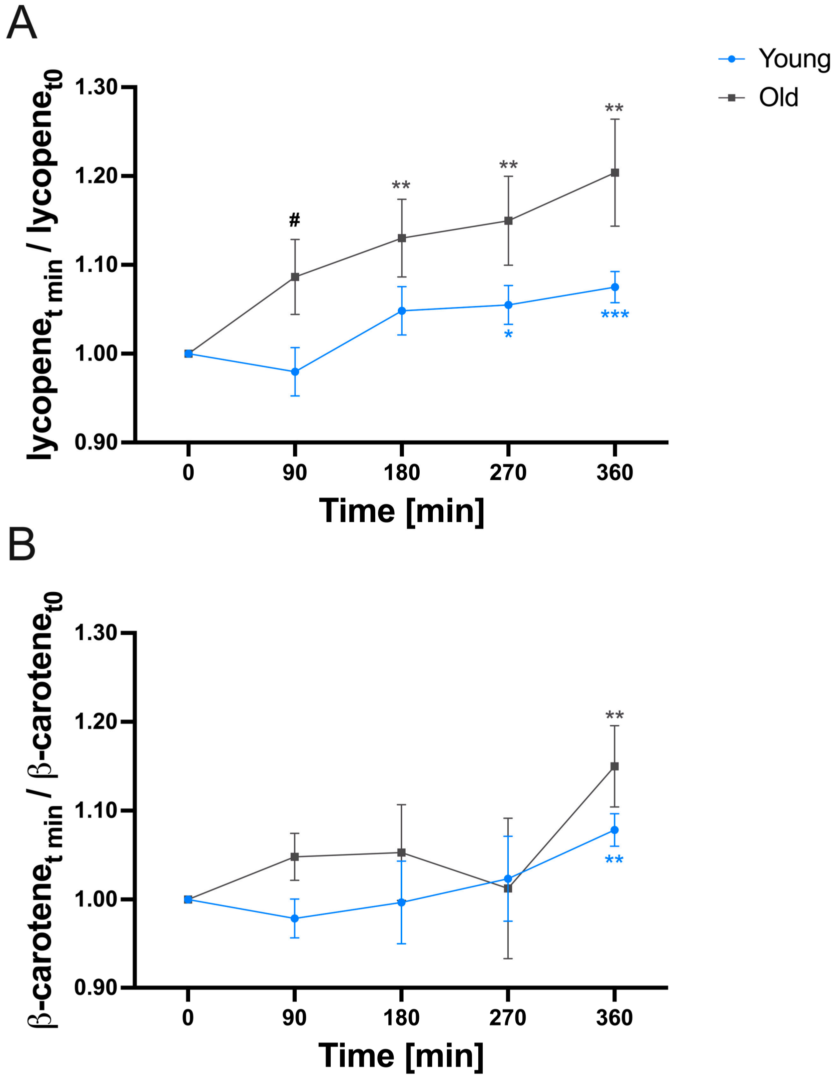

Figure S2. Postprandial progression curves of (A) lycopene and (B) β-carotene plasma concentrations throughout 360 min after consumption of a carotenoid-enriched supplement within the young (blue) and old (gray) study groups. Shown are the mean (± SEM) ratios of the serum concentrations between each observed timepoint and baseline to reflect postprandial variability. Significant time-dependent differences are marked with $p < 0.05$ (*); $p < 0.01$ (**); $p < 0.001$ (***). Significant differences that were age-related are marked with $p < 0.05$ (#). Calculations for significance were performed using repeated measurement two-way ANOVA followed by Fisher's LSD post-hoc test.

Exclusion criteria:

- Type 1 or type 2 diabetes
- Obesity (BMI ≥30 kg/m^2)
- Underweight (BMI < 19 kg/m^2)
- Regular intake of dietary supplements (multivitamins, carotenoids, trace elements) if subjects were not willing to abstain from them 3 days before intervention
- active smoking (≥ 4 cigarettes/day)
- Chronic diseases that affect gastrointestinal function
- pregnancy, lactation
- acute, active upper or lower respiratory tract infection
- drastic, unwanted weight loss (>5% in the last 6 months)
- (severe) food intolerances/allergies to tomatoes, carrots, herbs, soy, seafood/algae, gluten, nuts
- dementia
- self-reported severe/active diseases of the liver, kidney, rheumatic diseases and malignant tumors
- self-reported elevated blood fat levels (hyperlipidemia)
- Medications that influence fat absorption/metabolism/fat transport
- cardiac pacemakers

Supplemental Table S1

Table S1. Ingredients and quantities for the test meal.

Component	Ingredient	Quantity per portion
Bread Spread (30 g)	Mineral water non-carbonated	11.3 g (37.5 %)
	Carrots (hot air-fried	4.5 g (15.0 %)
	Tomatoes (dried)	3.8 g (12.5 %)
	Rapeseed oil	3.0 g (10.0 %)
	Sunflower seed paste	1.8 g (6.0 %)
	Hokkaido pumpkin (hot air-fried)	1.5 g (5.0 %)
	Wheat germ	1.4 g (4.5 %)
	Frozen chopped parsley	1.2 g (4.0 %)
	Frozen chopped chives	0.6 g (2.0 %)
	Sea buckthorn oil	0.6 g (2.0 %)
	Brazil nut	0.3 g (1.0 %)
	Table salt	0.1 g (0.3 %)
Smoothie (500 g)	Blackcurrant juice	130 g (26.0 %)
	Apple juice	130 g (26.0 %)
	Carrot juice	63.0 g (12.6 %)
	Cucumber	58.0 g (11.6 %)
	Tomato juice	31.0 g (6.2 %)
	Spinach, fresh	31.0 g (6.2 %)
	Sunflower seeds	22.5 g (4.5 %)
	Wheat germ	13.5 g (2.7 %)
	Parsley, fresh	8.0 g (1.6 %)
	Sea buckthorn juice	7.5 g (1.5 %)
	Lemon juice	6.0 g (1.2 %)

Supplemental Table S2

Table S2. Trace element concentrations for the test meal components and corresponding percentage proportions of the DRI according to the German Nutrition Society.

**Food item**	**Fe [mg]**		**Zn [mg]**		**Cu [mg]**		**Se [µg]**	
	Per serving	% DRI[a] m | f	Per serving	% DRI[b] m | f	Per serving	% DRI[c]	serving	% DRI[d] m | f
Margarine, 20 g	0.012	0.12 | 0.08	0.032	0.23 | 0.40	0.008	0.53	NDA	NDA
Wheat toast, 72 g	0.832	8.32 | 5.55	0.706	5.04 | 8.83	0.135	9.00	0.936	1.34 | 1.56
Smoothie, 500 g	2.466	24.7 | 16.4	2.989	21.4 | 37.4	0.360	24.0	14.6	20.9 | 24.3
Spread, 30 g	0.410	4.10 | 2.73	0.498	3.56 | 6.23	0.093	6.20	1.29	1.84 | 2.15
Sum	3.720	37.2 | 24.8	4.225	30.2 | 52.8	0.596	39.7	16.8	24.0 | 28.0

DRI: daily recommended intake; NDA: no data available

[a]male: 10 mg/d; female: 15 mg/d

[b]male: 14 mg/d; female: 8 mg/d (corresponds to recommendation for medium (660 mg/d) phytate intake)

[c]male/female: 1.5 mg/d

[d]male: 70 µg/d; female: 60 µg/d

Supplemental Table S3

Table S3. Sex-specific differences in examined parameters provided as mean ± SD.

	young male	old male	young female	old female	young vs. old (male)	young vs. old (female)	sex-specific differences (young)	sex-specific differences (old)
**Fe** [µg/L]	1568.0 ± 658.0	1677.0 ± 255.9	1173.0 ± 430.8	1288.0 ± 351.8	ns	ns	ns	ns
**Cu** [µg/L]	757.8 ± 153.7	845.4 ± 84.1	822.1 ± 203.9	918.7 ± 104.1	ns	ns	ns	ns
**Zn** [µg/L]	805.6 ± 30.7	720.0 ± 72.4	686.4 ± 70.5	657.2 ± 51.99	*	ns	***	ns
**Se** [µg/L]	74.47 ± 6.78	73.31 ± 9.88	66.34 ± 13.50	80.49 ± 11.07	ns	*	ns	ns
**I** [µg/L]	55.56 ± 9.46	56.33 ± 5.50	54.52 ± 8.05	57.33 ± 9.33	ns	ns	ns	ns
**Cu/Zn ratio**	0.98 ± 0.22	1.19 ± 0.18	1.20 ± 0.31	1.37 ± 0.18	ns	ns	ns	ns
**Se/Cu ratio**	0.097 ± 0.023	0.088 ± 0.016	0.086 ± 0.030	0.088 ± 0.014	ns	ns	ns	ns
**fZn** [nM]	0.57 ± 0.13	0.74 ± 0.20	0.68 ± 0.15	0.59 ± 0.14	ns	ns	ns	ns
**retinol** [µmol/L]	1.463 ± 0.150	1.572 ± 0.366	1.382 ± 0.322	1.542 ± 0.284	ns	ns	ns	ns
**vitamin D** [nmol/L]	65.38 ± 25.48	81.51 ± 21.45	72.05 ± 28.62	139.9 ± 46.86	ns	***	ns	**
**vitamin C** [mmol/L]	0.190 ± 0.105	0.204 ± 0.198	0.664 ± 0.804	0.278 ± 0.202	ns	ns	ns	ns
**lycopene** [µmol/mmol cholesterol]	0.173 ± 0.037	0.126 ± 0.033	0.159 ± 0.055	0.107 ± 0.025	ns	ns	ns	**
**β-carotene** [µmol/mmol cholesterol]	0.103 ± 0.040	0.071 ± 0.027	0.542 ± 0.271	0.147 ± 0.056	ns	***	***	ns

Statistical analyses by ordinary one-way ANOVA with Tukey's multiple comparisons; $p < 0.05$ (*); $p < 0.01$ (**); $p < 0.001$ (***)
